# Effect of prolonged submaximal exercise on serum oxidative stress biomarkers (d-ROMs, MDA, BAP) and oxidative stress index in endurance horses

**DOI:** 10.1186/s12917-018-1540-y

**Published:** 2018-07-06

**Authors:** Nika Brkljača Bottegaro, Jelena Gotić, Jelena Šuran, Diana Brozić, Karla Klobučar, Krunoslav Bojanić, Zoran Vrbanac

**Affiliations:** 1 Clinic for Surgery, Orthopaedics and Ophthalmology, Faculty of Veterinary Medicine, University of Zagreb, Heinzelova 55, 10 000 Zagreb, Croatia; 2 Clinic for Internal Diseases, Faculty of Veterinary Medicine, University of Zagreb, Heinzelova 55, 10 000 Zagreb, Croatia; 3 Department of Pharmacology and Toxicology, Faculty of Veterinary Medicine, University of Zagreb, Heinzelova 55, 10 000 Zagreb, Croatia; 4 Department of Animal Nutrition and Dietetics, Faculty of Veterinary Medicine, University of Zagreb, Heinzelova 55, 10 000 Zagreb, Croatia; 5 Faculty of Veterinary Medicine, University of Zagreb, Heinzelova 55, 10 000 Zagreb, Croatia; 6 School of Veterinary Science, Massey University, Palmerston North, New Zealand; 7 Department of Radiology, Ultrasound Diagnostic and Physical Therapy, Faculty of Veterinary Medicine, University of Zagreb, Heinzelova 55, 10 000 Zagreb, Croatia

**Keywords:** Horse, Oxidative stress, Reactive oxygen metabolites, Malondialdehyde, Biological antioxidant potential, Oxidative stress index, Endurance race

## Abstract

**Background:**

Oxidative stress (OS) associated with an intense exercise may have a negative influence on equine health. The aim of this study was to determine the effects of endurance races on oxidative and antioxidative status of horses by evaluating changes in reactive oxygen metabolites (d-ROMs), malondialdehyde (MDA), biological antioxidant potential (BAP) and oxidative stress index (OSI) values. The study was carried out on 53 race starts (28 individual horses) competing at different endurance races according to distance (40 and 80 km) and difficulty (easy and demanding). Blood samples were taken before and after the race.

**Results:**

Compared to levels of OS serum biomarkers before the race, an increase in values of d-ROMs (*P* <  0.01), MDA (*P* <  0.01), and BAP (*P* <  0.001), and a decrease in OSI (*P* <  0.001) have been noted after the race. Contrary to other measured biomarkers, BAP did not show significant individual effects of horses. Horses competing at shorter races have shown a significant change in d-ROMs (*P* = 0.002), BAP (*P* <  0.001) and OSI (*P* = 0.004), whereas those competing at longer races in MDA (*P* = 0.002), BAP (*P* <  0.001) and OSI (*P* <  0.001) post-race values. Endurance racing induced changes in values of d-ROMs, BAP and OSI during both easy and demanding races.

**Conclusions:**

Changes in all measured OS biomarkers indicate that prolonged aerobic exercise during endurance race could contribute to the imbalance between oxidants and antioxidants in horses, mainly characterised by a pronounced antioxidant response. Biological antioxidant potential was found to be the most reliable biomarker of OS in endurance horses in the present study.

## Background

Endurance riding is an extremely demanding equine discipline since horses compete over distances of up to 160 km in a day. An endurance ride is divided into several phases of 20 to 40 km, each followed by a mandatory resting period. Before the ride, as well as after each phase, horses undergo a mandatory veterinary examination and can be eliminated if their metabolic status or orthopaedic condition are found inadequate to enable them to continue the ride.

Exercise induces increased oxygen consumption which in return affects the oxidant/antioxidant status [5]. During short-term maximal exercise in Thoroughbred racehorses, there is an increase in oxygen consumption that reaches levels 30 times above basal [1]. Even though endurance riding is classified as a prolonged aerobic exercise [2], the high oxygen demands over a longer period of time result in an increased reactive oxygen species (ROS) formed by 1 to 2% of the oxygen that is not completely reduced into carbon dioxide and water [3]. The consequence of increased oxidants is a disarranged equilibrium between oxidants and antioxidants resulting in oxidative stress (OS) [4]. Apart from chronic accumulation of ROS, OS can also be a sequel of insufficient antioxidant defence systems, causing damage to all cell components, especially DNA, lipids, and proteins [5].

OS is characterised by changes in different biomarkers but the determination of reactive oxygen metabolites (d-ROMs), an indirect method evaluating the free radicals in the serum, is considered a ‘gold standard’ biomarker for measuring total systemic oxidative status [6]. Among other OS biomarkers, the biological antioxidant potential (BAP) test indicates systemic antioxidative properties and reflects the reduction potential in the serum [7], whereas malondialdehyde (MDA) is one of the better-known secondary products of lipid peroxidation [8] used as an indicator of cell membrane injury [9]. The oxidative stress index (OSI) is an arbitrary parameter derived from values of d-ROMs and BAP to represent an individual’s antioxidant potential against the condition of OS [10]. In equine medicine, OSI has been investigated in resting Thoroughbred foals [11], Thoroughbred racehorses [12], and Thoroughbred horses after castration [13].

Oxidative stress in horses induced by endurance races has been investigated in a few studies mainly focusing on antioxidant status [14–17]. Oxidative properties have been assessed by measurements of the following biomarkers: lipid hydroperoxides (LPO) [17, 18] and total barbituric acid reactive substances (TBARS) [14, 16]. On the other hand, antioxidant status in endurance horses has been more intensively studied by evaluating several different biomarkers: α-tocopherol (TOC) and glutathione (GSH) [15–18]; glutathione peroxidase (GPx) activity [14, 15, 17, 18], glutathione redox ratio [16], glutathione reductase, superoxide dismutase (SOD), and total antioxidans status (TAS) [14].

However, data on changes of d-ROMs and BAP in relation to endurance exercise in horses, to the best of our knowledge, have not been reported. All the mentioned studies [14–18] have been performed on horses competing at only higher levels of exercise, although a vast majority of horses in this sport compete at lower level national endurance races.

The aim of this study was to determine the effects of endurance races on blood levels of d-ROMs, BAP, and MDA OS biomarkers in horses. It was hypothesised that even endurance races at shorter distances induce OS in horses. The second hypothesis was that endurance exercise causes changes in OSI levels which could emphasise a more pronounced prooxidant or antioxidant response.

## Methods

### The competition and conditions

The study included horses competing at four national competitions held from April to July 2017 at different locations in Croatia. The horse owners were offered to participate in the study prior to the first veterinary inspection of their horse (s) at the competition sites. The races differed according to terrain condition and configuration, altitude changes, and weather conditions, thus considering these variations, the races were categorised as demanding or easy. Demanding races were those with a considerable part of rocky racetrack, higher altitude differences and/or with higher temperature and humidity. Two races were classified as easy and two as demanding. Also, there were races of both 40 and 80 km distances at each competition.

### Horses

All horses competing at the mentioned races were eligible for enrolment in this study. Forty-five horses were enrolled following owner consent. Horses had been transported from varying distances and arrived at the event several hours (at least two) before the first veterinary examination. All the horses participating in the study successfully passed the veterinary inspection prior the start, as well as all the inspections during and after the race. Since the inclusion criterion for horses was to successfully complete the race, only the finisher horses were included in the further study.

The average speed of horses in the study population was also evaluated with a cut-off of 14 km/h which is the minimal average speed required for the Federation Equestre International 4* championship qualification according to the FEI Endurance rules 2017.

### Sample collection and analysis

Blood samples (5 ml) were collected by jugular venipuncture into vacuum tubes for serum with gel using a vacutainer (Vacutainer®, Becton Dickenson, USA) 1 h before the start and 30 min after the end of the race. The samples were left to coagulate for 30 min and thereafter centrifuged at 3500 pm for 15 min. The samples were shipped in cooling bags at 4 °C to the laboratory, and within 4 h after sampling stored at − 80 °C until further analyses.

Serum levels of d-ROMs and BAP were measured using Diacron® commercial kits (Grosseto, Italy) on biochemical analyzer Olympus AU640 (Olympus, Japan) according to manufacturer’s instructions. The results of the d-ROMs analysis were expressed in Carratelli units (U.Carr.), where 1 U.Carr. corresponds to 0.8 mg/L H_2_O_2_. The results of the BAP analysis were expressed in μmol/L of the reduced ferric ions. The assays used in this study had been previously validated for use in horses [6, 19].

Malondialdehyde concentration was determined with a slight modification of a previously reported method [8]. Briefly, a volume of 150 μL of serum was added to 50 μL of water and 50 μL of NaOH (3 N). After 30 min of shaking water bath with an incubation at 60 °C, 250 μL 6% phosphoric and 0.8% thiobarbituric acid were added and the mixture was heated at 90 °C for 45 min. The mixture was cooled and extracted by adding 250 μl of pure methanol and 100 μl 10% of sodium dodecyl sulphate, vortex-mixed for 1 min and centrifuged at 3000×g for 10 min. A Shimadzu 2010 LC system equipped with an InertSustain C18 (4.6 mm × 150 mm × 5 μm) column (GL Sciences) and a UV–visible detector was used for analysis with the following conditions: the UV wavelength was set at 532 nm, injection volume of 20 μL, and the column oven temperature at 38 °C. The mobile phase was as follows: pump A, methanol, and pump B, 50 mM potassium dihydrogen phosphate with pH of 6.2 (50:50, *v*/v). The flow rate of the mobile phase was 1 mL/min with constant flow control. The standard curve was prepared using 1,1,3,3-tetraethoxypropane with the linearity of 0.98 and reliability of 97.5% for given standard concentration range (0.5–15.175 μM of standard). MDA concentration was expressed as μmol per L serum. The method was in-house validated for use in horse serum, with the inter- and intra-assay coefficients of variation of less than 5%.

The OSI was calculated from the measured values of d-ROMs and BAP using the ratio of d-ROMs/BAP multiplied by 100 [11].

### Statistical methods

All exploratory data analyses and statistical tests were performed using the R programming language version 3.2.21 [20]. The dependent variables of interest were the changes in values after the race compared to before the race for each OS biomarker. The explanatory variables of interest that were collected from horses (gender and average speed) and race events characteristics (race distance and difficulty) were used to evaluate the changes in values of OS biomarkers within each subpopulation category and for comparisons between the respective subpopulation categories of horses. Horses never ran more than once per event and some horses participated at several events. Therefore, the overall average change (post-race value – pre-race value) was analysed via hierarchical linear mixed-effects regression models due to repeated measurements on the same horse subjects and using linear regression models for analysis of data within each race. Modelling was performed using the lme4 software package [21]. Outliers were assessed using leave-one-out method for mixed models and Bonferroni outlier test for linear models. As no significant influence on model coefficient estimates was observed, the data were kept in the model. The alpha level of significance for model fits and regression coefficients was taken at less than 0.05. Data were expressed as an average (±standard error of mean (SEM)).

## Results

### Study population characteristics

Forty-five horses participated in the study. However, 28 individual horses successfully met the inclusion criteria in 53 race starts, since some horses participated in more races. The characteristics of the study population are presented in the Table 1. The average age of the studied horses was 7.3 years (±0.3 SEM) and their average speed during races was 13.5 (±0.2 SEM) km/h. The average speed was 13.7 (±0.3 SEM) km/h at 40 km races and 13.2 (±0.3 SEM) km/h at 80 km races. There were no significant differences between the average speed at 40 and 80 km races (*P* = 0.3). However, there was a significant association (*P* <  0.001) of the average speed and the race difficulty with the average speed of 14.3 (±0.2 SEM) km/h at easy races and the average speed of 12.3 (±0.4 SEM) km/h at demanding races.

**Table 1:** Characteristics of the study population of horses attending four endurance races

Descriptive variables	Study population
Horses	28 individuals
Breed (Arabian / other breeds)	14 / 14
Gender (females / males)	18 / 10
Races	53 records
Distance (40 km / 80 km)	30 / 23
Race difficulty (easy/demanding)	32 / 21
Average speed (< 14 km/h / ≥ 14 km/h)	35 / 18
Races per horse (one / two / three / four races)	8 / 15 / 5 / 0

### Comparison of pre- and post-race values of OS biomarkers

**Fig. 1:**
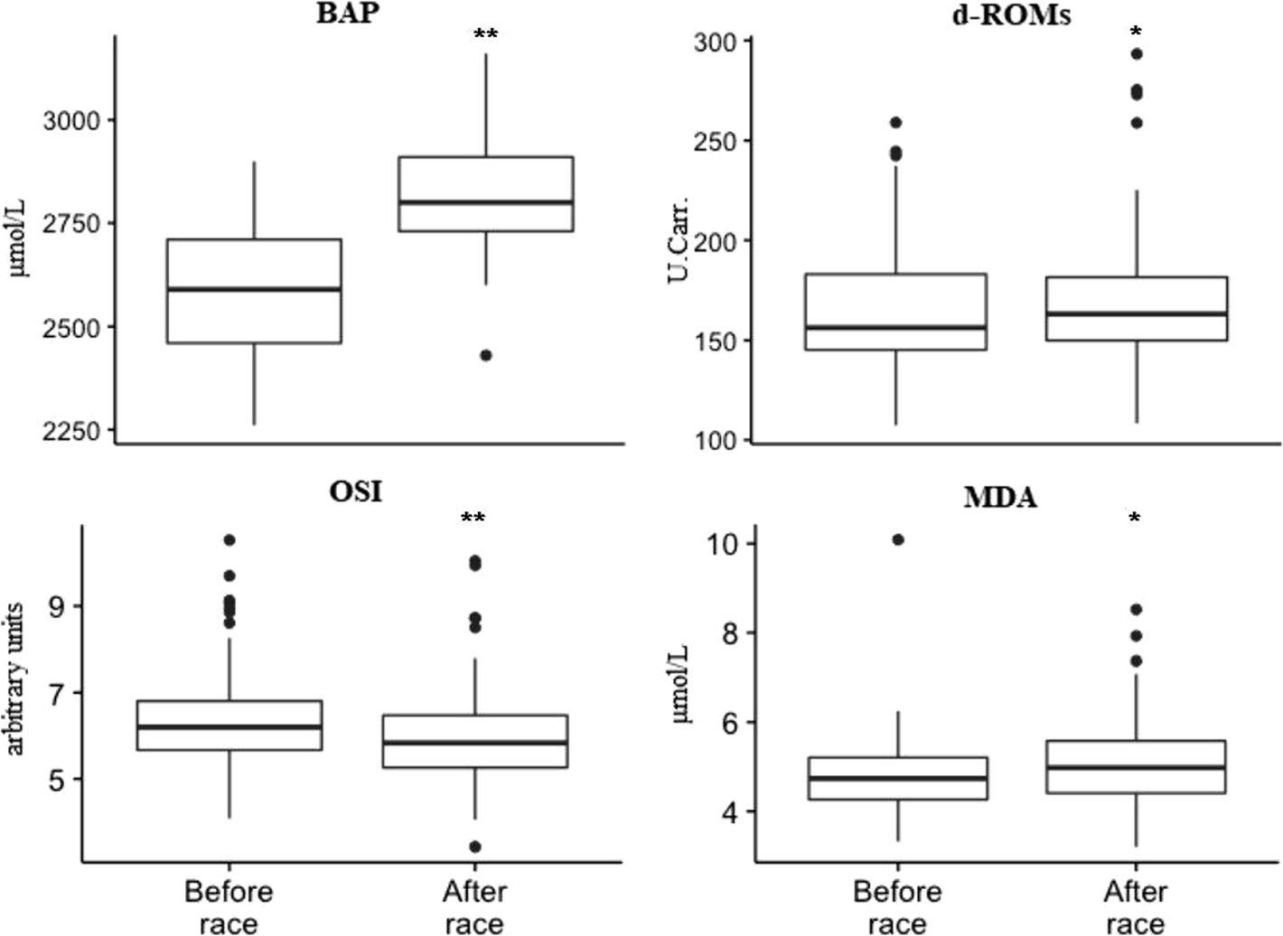
Boxplots of values of the OS biomarkers in endurance horses pre- and post-races. The data represents measurements from 53 race runs of 28 individual horses over four racing events. The null hypothesis states there is no difference between the two (i.e., the delta change being zero). Significant differences from values at baseline (before race); *P <  0.01; **P <  0.001; d-ROMs, determination of reactive oxidative metabolites; BAP, biological antioxidant potential; MDA, malondialdehyde; OSI, oxidative stress index

The values of all oxidative stress markers evaluated in this study have significantly changed after the race compared to the values before the race and their distribution is depicted in Fig. 1. The most significant changes were noted for BAP and OSI (*P* <  0.001). On average, values of BAP, d-ROMs, and MDA increased, while values of OSI decreased after the race. Inspection of random effects of horse subjects showed that the estimates of average change of MDA, d-ROMs, and OSI are extremely variable between and within horses. Ten horses had discordant changes (an increase at one race and a decrease at another) in values of d-ROMs, 11 in MDA and 8 in values of OSI. On the other hand, the individual variation of the subjects was low for BAP.

Values of different OS markers in relation to categories of race distances and difficulty, and of average speed and gender of horses are presented in Tables 2 and 3, respectively. On average, there was a significant change in values of BAP and OSI irrespective of the race distances. However, a significant change in values of d-ROMs was observed only at 40 km and of MDA only at 80 km race distances. Values of d-ROMs, BAP and OSI showed significant differences before and after the race for both easy and demanding races. Changes in concentrations of MDA were significantly different than before the race only after easy races. Dividing the horse population in accordance with the average speed obtained, the changes in levels of d-ROMs increased significantly after the race only in horses of the faster speed category, levels of BAP increased significantly in both speed categories, and the concentration of MDA and values of OSI had significant changes only in the slower speed category. Both female and male horses showed significant differences in values of d-ROMs, BAP, and OSI after the race, compared to the values before the race. However, a significant change in concentrations of MDA due to race has been noted just in female horses. Both race distance and difficulty had a significant association with the change of BAP. The changes in values of OSI before and after the race have been influenced by race distance and average speed of horses.

**Table 2:** Values of serum OS biomarkers in horses pre- and post-races by distance and difficulty

Parameter (unit)		Distance		Race difficulty	
		40 km	80 km	Easy	Demanding
d-ROMs (U.Carr.)	Pre-race	164.8 ± 7.6	168.7 ± 9.9	166.2 ± 6.8	166.2 ± 8.2
	Post-race	173.8 ± 8.6	174.2 ± 11.3	171.9 ± 7.8	172.3 ± 8.4
	P	0.002	0.2	0.04	0.03
	P^a^	0.5		0.9	
BAP (μmol/L)	Pre-race	2610.9 ± 33.2	2533.1 ± 30.5	2612.7 ± 32.2	2541.9 ± 32.2
	Post-race	2820.4 ± 28.2	2840.4 ± 31.4	2817.5 ± 28.8	2840.5 ± 22.8
	P	< 0.001	< 0.001	< 0.001	< 0.001
	P^a^	0.01		0.02	
MDA (μmol/L)	Pre-race	5.1 ± 0.2	4.5 ± 0.1	4.7 ± 0.1	5.1 ± 0.3
	Post-race	5.3 ± 0.2	4.7 ± 0.2	5.1 ± 0.2	5.1 ± 0.3
	P	0.1	0.02	0.003	0.7
	P^a^	0.9		0.1	
OSI (arbitrary units)	Pre-race	6.3 ± 0.3	6.7 ± 0.4	6.4 ± 0.3	6.6 ± 0.3
	Post-race	6.1 ± 0.3	6.2 ± 0.4	6.1 ± 0.3	6.1 ± 0.3
	P	0.004	< 0.001	0.005	< 0.001
	P^a^	0.01		0.1	

*d-ROMs* determination of reactive oxidative metabolites, *BAP* biological antioxidant potential, *MDA* malondialdehyde, *OSI* oxidative stress index

P-significance for the differences within each category division (e.g. change in values at 40 km and at 80 km separately)

P^a^-significance for the difference between the two categories (e.g. change in values at 40 km vs. at 80 km)

**Table 3:** Values of serum OS biomarkers in horses pre- and post-races by average speed and gender

Parameter (unit)		Average speed		Gender	
		< 14 km/h	≥ 14 km/h	Female	Male
d-ROMs (U.Carr.)	Pre-race	166.5 ± 7.3	160.6 ± 8.2	171.1 ± 8.0	164.1 ± 11.9
	Post-race	171.5 ± 8.2	170.1 ± 8.9	177.2 ± 9.8	170.2 ± 11.8
	P	0.08	0.002	0.03	0.03
	P^a^	0.3		0.8	
BAP (μmol/L)	Pre-race	2570.2 ± 28.4	2585.4 ± 39.6	2570.8 ± 29.82	2588.5 ± 46.9
	Post-race	2849.2 ± 23.3	2787.5 ± 41.0	2816.3 ± 21.3	2825.2 ± 51.5
	P	< 0.001	< 0.001	< 0.001	< 0.001
	P^a^	0.1		0.9	
MDA (μmol/L)	Pre-race	4.7 ± 0.1	5.2 ± 0.3	4.8 ± 0.1	4.9 ± 0.3
	Post-race	5.0 ± 0.2	5.3 ± 0.3	5.1 ± 0.1	5.1 ± 0.4
	P	0.003	0.3	0.01	0.2
	P^a^	0.8		0.9	
OSI (arbitrary units)	Pre-race	6.5 ± 0.3	6.2 ± 0.3	6.7 ± 0.4	6.4 ± 0.5
	Post-race	6.04 ± 0.3	6.1 ± 0.4	6.4 ± 0.5	6.4 ± 0.5
	P	< 0.001	0.116	< 0.001	< 0.001
	P^a^	0.02		0.6	

*d-ROMs* determination of reactive oxidative metabolites, *BAP* biological antioxidant potential, *MDA* malondialdehyde, *OSI* oxidative stress index

P-significance for the differences within each category division (e.g. change in values at female and at male horses separately)

P^a^-significance for the difference between the two categories (e.g. change in values at female vs. male horses)

Table 4 presents values of different OS markers in relation to each of the four races. Values of d-ROMs showed significant differences before and after the race during 40 km at the first race. With regard to values of BAP, significant differences before and after the race values have been noted in all but the second race. In addition, the changes in values of BAP were more pronounced after 80 km than after 40 km races at the third and fourth race events. Values of OSI showed significant differences before and after the race during 80 km at the third and after 40 km at the fourth race event.

**Table 4:** Values of serum OS biomarkers in horses pre- and post-races by separate race events

Parameter (unit)		First race		Second race		Third race		Fourth race	
		40 km (*n* = 12)	80 km (*n* = 7)	40 km (*n* = 2)	80 km (*n* = 3)	40 km (*n* = 8)	80 km (*n* = 8)	40 km (*n* = 8)	80 km (*n* = 5)
d-ROMs (U.Carr.)	Pre-race	160.9 ± 11.8	172.7 ± 16.8	163.8 ± 13.5	184.9 ± 27.4	162.7 ± 9.2	163.3 ± 17.5	174.9 ± 6.2	157.2 ± 12.1
	Post-race	176.0 ± 13.2	175.6 ± 20.2	161.7 ± 0.7	184.2 ± 22.0	169.1 ± 8.9	173.8 ± 19.7	173.0 ± 8.2	156.7 ± 14.7
	P	< 0.001	0.7	0.9	0.9	0.06	0.07	0.7	0.9
	P^a^	0.07		0.9		0.5		0.9	
BAP (μmol/L)	Pre-race	2575.8 ± 51.6	2628.6 ± 71.9	2820.0 ± 40.0	2573.3 ± 53.6	2556.3 ± 52.6	2446.3 ± 21.5	2692.5 ± 34.3	2516.0 ± 53.5
	Post-race	2806.7 ± 49.0	2837.1 ± 90.3	2905.0 ± 155.0	2883.3 ± 69.4	2812.5 ± 33.6	2836.3 ± 32.3	2818.8 ± 29.3	2826.0 ± 65.7
	P	< 0.001	0.04	0.7	0.06	< 0.001	< 0.001	< 0.01	< 0.01
	P^a^	0.8		0.3		0.03		0.02	
MDA (μmol/L)	Pre-race	4.9 ± 0.2	4.5 ± 0.2	8.2 ± 1.9	3.8 ± 0.2	5.2 ± 0.2	4.6 ± 0.1	4.8 ± 0.1	4.3 ± 0.2
	Post-race	4.9 ± 0.2	4.9 ± 0.3	7.8 ± 0.7	3.5 ± 0.1	5.3 ± 0.2	4.8 ± 0.2	5.7 ± 0.4	5.2 ± 0.3
	P	0.8	0.2	0.8	0.08	0.5	0.5	0.08	0.02
	P^a^	0.3		0.9		0.9		0.9	
OSI (arbitraryunits)	Pre-race	6.2 ± 0.4	6.7 ± 0.8	5.8 ± 0.4	7.1 ± 0.9	6.4 ± 0.4	6.7 ± 0.7	6.5 ± 0.3	6.2 ± 0.4
	Post-race	6.2 ± 0.4	6.3 ± 0.9	5.6 ± 0.3	6.4 ± 0.7	6.0 ± 0.3	6.1 ± 0.6	6.1 ± 0.3	5.5 ± 0.5
	P	0.9	0.07	0.2	0.07	0.08	0.02	0.01	0.2
	P^a^	0.05		0.1		0.4		0.4	

*d-ROMs* determination of reactive oxidative metabolites, *BAP* biological antioxidant potential, *MDA* malondialdehyde, *OSI* oxidative stress index

P-significance for the differences within each category division (e.g. change in pre-race and post-race values)

P^a^-significance for the difference between the two categories (e.g. change in values at 40 km vs. 80 km)

## Discussion

The intense exercise during endurance races in this study induced OS in horses demonstrated by increased values of d-ROMs, MDA, and BAP, and a decreased values of OSI. The increase in values of d-ROMs and MDA in horses undergoing endurance races could be attributed to the enhanced production of free radicals and lipid peroxidation resulting in oxidative damage [6, 22]. On the other hand, the rise in levels of BAP after the race indicates a mobilization of antioxidants as a response to an increased amount of ROS [6]. Oxidative stress depends on the balance between ROS and body’s antioxidant system [23], therefore, OSI could more accurately reflect OS status due to a joint measure of oxidants and antioxidants properties. In the present study, a significant decrease in values of OSI shown after the race indicates a pronounced antioxidant response induced by endurance exercise.

Previous studies have evaluated OS in response to endurance exercise. Endurance race of 80 km caused an effect on oxidative status balance characterised by an increase in values of LPO, GSH and a decrease in values of GPx and TOC [17]. A study [18] reported OS after 80 km endurance race by an increase in total plasma LPO. On the other hand, changes in oxidative status markers (lipid hydroperoxides oxygen radical absorbance capacity, vitamin E, and GSH) have not been observed after 80 km endurance race [24]. The discrepancy between the mentioned findings [24] and the present study could be attributed to OS biomarker selection, a small number of horses (four), and a relatively low average speed of 10.5 km/h in the former compared to the present study which had a larger sample size and horses competing with almost 30% faster average speed. A previous report [2] assessed the levels of SOD, GPx, reduced GSH, catalase, and the plasma MDA in endurance horses, performing at different distances. Interestingly, that study did not record any significant changes in values of OS biomarkers after the race, although higher values of SOD, GPx, GSH, and MDA were recorded after the 80 km race but not at 160 km. These results could also be attributed to the relatively small number (15) of studied horses and potentially individual differences in measured OS biomarkers could have precluded the true association of OS biomarkers and race distance. As the effect of individual horses could vary between OS biomarkers, the selection of the markers should be an important aspect for consistent results. The results of the present study showed only BAP not to have a significant horse effect, hence, making it a more consistent OS biomarker than d-ROMs, MDA, and OSI. In this study d-ROMs, MDA, and OSI showed discordant changes in approximately one-third of the horses. These findings indicate that there are other determinants and explanatory variables also influencing the changes in d-ROMs, MDA, and OSI that have not been followed in this study. Similarly, a report [25] noted fluctuation of values of d-ROMs especially, but also of BAP to a lesser degree, in ten Thoroughbred horses. Studies involving a large number of horses are needed in order to better understand the physiological levels and changes of OS biomarkers.

In the present study, MDA has also shown increased concentrations after the race indicating a potential cumulative cell membrane lipid peroxidation. A rise in erythrocyte MDA concentration was also recorded after short-term heavy exercise in jumping horses [22], as well as by an increased intensity ride in ten Maremmana stallions [26]. A previous study [27] reported significant changes of TBARS, a test that measures mainly but not only MDA, induced at 80 km endurance race, but not in horses competing at 160 km. That study suggested horses competing at higher speed (which occurred at 80 km races) are more prone to rise in TBARS levels after exercise. Contrary, a study on five horses competing at 160 km race, noted an increase in values of TBARS after the race [14]. In the present study, an increased concentration of MDA was noted only in horses at 80 km races with no significant difference in average speed between race distances thus, it is not possible to confirm the suggestion of influence of speed [27]. Lack of increased lipid peroxidation after half race distance and an increase after 80 km endurance race have also been noted by measuring values of plasma LPO [17]. Actually, the present study showed a significant rise in the concentration of MDA after the race only at slower average speed. Furthermore, the present study also adds race difficulty as another factor potentially influencing changes of MDA, as a significant change was shown only at easy races. These unexpected results raise suspicion for a potential individual variation in lipid peroxidation between horses and the possibility that training status or other parameters not followed in this study, could influence concentrations of MDA. On the other hand, lower values of LPO have been recorded in an easier race in comparison to a more difficult race as defined by the riders [17]. However, a clear definition of race difficulty could be challenging, especially considering possible subjectivity of riders opinion.

In previous studies of OS subsequent endurance races, opponent changes in antioxidant status have been noted. After 80 km race an increase in values of GSH [17, 18] and GPx [15, 18] has been noted, contrary, another study showed a decrease in values of GPx [17]. A study on 5 horses competing at 160 km endurance race noted a drop in values of TAS at 80 km, but a rise of both values of SOD and TAS at the race end [14]. The inconsistent findings of previous studies could be attributed to the antioxidant biomarker selection, since some of the vast antioxidant biomarkers show lower antioxidant capacity caused by exercise, but simultaneously other biomarkers enhance their activity. Therefore we believe that BAP could be more effective in delineating general antioxidant status, since it provides an overall measurement of different antioxidants including uric acid, ascorbic acid, proteins, TOC, bilirubin and others [7]. The increase in levels of BAP after the race in the present study indicates an increase in the antioxidant potential as a consequence of exercise. It suggests that during prolonged exercise OS induces a response to the increase in ROS thus, results in an increase in levels of BAP. Similar findings have also been noted in Thoroughbred horses after a short-term maximum treadmill exercise [28]. In that study, the rise in BAP levels was more pronounced immediately after exercise, with a clear drop in levels of BAP 30 min after exercise. After finishing the race endurance horses have a maximum of 30 min rest before presenting to the final inspection. Owners did not allow blood sampling during that rest period in the present study since it could negatively affect the horse’s attitude thus compromising the final results of the forthcoming mandatory veterinary inspection. Therefore, our sampling time was 30 min after the end of the race and it could have been the reason of our comparably lower recorded levels of BAP, although still significantly different from the values before the race. Similarly, in the study were samples have been taken after 80 km race as well as after 60 min of recovery period, there was an evident decrease in GPx levels during the last sampling [15]. Though, in the abovementioned study it is not defined if the samples have been taken immediately after finishing the race or after passing the mandatory veterinary inspection. The time preceding the veterinary inspection after each loop is very short (often lasting up to few minutes) and considerable effort is put on minimising any discomfort to horses. In the study on Thoroughbred horses undertaking treadmill exercise [28], an increase in levels of d-ROMs in response to exercise has been reported. Interestingly, in that study, at 30 min after exercise, the levels of d-ROMs returned to baseline. Therefore, the sampling time could have also influenced the results of d-ROMs in the present study. Similarly, in a previous study an effect of 3 h recovery period after 80 km endurance race on prooxidative biomarker values has been noted since values of LPO also decreased to baseline [17]. An overall increase in levels of d-ROMs in the present study has been noted after the race but, taking into account race distances, this increase was significant only in horses competing at shorter distances. Horses competing at 80 km races were running twice longer and furthermore had one more compulsory rest period according to endurance rules that could have influenced our results. In addition, the lack of the elevation of d-ROMs after 80 km races could also indicate that the horses used in this study had an adaptive antioxidant response. Horses competing at 80 km are trained for longer periods than those competing at 40 km what could induce adaptations to OS, supported by a study in Standardbred horses that proved that training increase antioxidant capacity [29].

In the present study, a decrease in values of OSI has been noted after the race similarly to a previous report on Thoroughbred horses [28] using the BAP/d-ROMs ratio, which is an inverse formula of the one used in the present study. Nevertheless, the results of both studies indicate a more pronounced antioxidant status as a result of OS induced by exercise. Reference ranges of d-ROMs, BAP and OSI values have been studied in 372 Thoroughbreds racehorses [12]. That study measured values of OS biomarkers only during resting and the levels of both BAP and d-ROMs OS markers have been very similar to our findings.

The observed changes in levels of BAP after exercise were associated with both race distance and difficulty with higher values recorded on longer and demanding races. These findings indicate that the type and intensity of exercise can also influence OS in endurance horses. In a previous study, comparing results between two 80 km races of unequal difficulties, a more pronounced rise in GSH has also been recorded after a more difficult race [17]. More pronounced changes in antioxidant biomarkers were noted in eventing horses competing at a higher intensity exercise indicating the influence of increased anaerobic and musculoskeletal demands [30]. Expectably, a more pronounced increase in levels of BAP coupled with a more pronounced decrease in values of OSI was recorded on longer compared to the shorter races, as well as on demanding compared to easy races, which could indicate a stronger antioxidant response due to a higher intensity of exercise. However, even when considering only horses participating at 40 km races an increase in values of BAP and OSI was evident after the race, which emphasises that horses competing at lower distances are also susceptible to OS, especially characterised by a pronounced activation of antioxidant defence mechanisms. Accordingly, OS could still have negative consequences on performance and health in shorter races.

Taking into account the speed of horses, more pronounced changes in values of OSI have been noted at slower average speed. Since less experienced horses often compete at a lower speed, this result could indicate that those horses are more susceptible to OS than more experienced horses. The same was proved in mice chronically exposed to exercise, that showed less oxidative damage after exhaustive exercise than the untrained animals. This is largely due to the upregulation of endogenous antioxidant enzymes such as mitochondrial SOD, GPx, and γ-glutamylcysteine synthetase [31]. On the other hand, more pronounced adaptation to OS in horses competing at higher speed could be explained by more intense regular training. An adaptive response to training has also been noted in serum amyloid A concentrations in endurance horses after training session resembling a race [32].

The results did not show gender to influence any studied OS markers, however, when separately evaluating female and male horses, only concentrations of MDA was significantly different from pre-race values in mares only. This could be a spurious finding due to a lesser number of male horses. A previous study on Warmblood horses noted increased SOD activities in response to exercise only in mares [33].

When comparing individually the 4 races results with the whole testing population, an inconsistency is present, especially in the second race. However, there were only 2 horses competing at 40 km and 3 horses competing at 80 km at the second race. Nevertheless, we find noteworthy mentioning that the BAP levels in all races, except the second race, have shown consistent results what additionally accentuates the reliability of this biomarker in representing antioxidative status in endurance horses.

There were some limitations of this study that need to be emphasised. Lack of standardised feeding or detailed nutritional surveys to determine the intake level of antioxidants can be considered a limitation. However, most of the horses were fed balanced formulas for endurance horses. In light of standardisation, as this study was not performed in an experimentally controlled setting, the heterogeneity of horses may have introduced bias or confounding of the results. Since all horses have been transported to the event from different locations, the transport per se could have also influenced the results despite that all the horses have been given a period of rest in proportion to the distance and stress caused by the transportation before the start of the competition. However, it was taken that the transportation effect had ceased since all the animals have successfully passed the veterinary examination and were declared fit to compete prior to the blood sampling. Another limitation was that the enrolment into the study was voluntary and it did not include all the participating horses.

## Conclusions

The submaximal endurance exercise-induced simultaneous changes in all measured OS markers in this study. Unlike d-ROMs, MDA, and OSI biomarkers, BAP was observed to have low individual variations between and within horses, making it the most reliable biomarker of OS in endurance horses in the present study. Our results indicate that disequilibrium between oxidants and antioxidants also occurred during shorter races and those performed on less demanding racetracks, both in particular characterised by a pronounced antioxidant defence.

## Abbreviations

BAP: Biological antioxidant potential
d-ROMs: Reactive oxygen metabolites
GPx: Glutathione peroxidase
GSH: Total glutathione
MDA: Malondialdehyde
OS: Oxidative stress
OSI: Oxidative stress index
ROS: Reactive oxygen species
TBARS: Total barbituric acid reactive substances
